# Emersion and Terrestrial Locomotion of the Northern Snakehead (*Channa argus*) on Multiple Substrates

**DOI:** 10.1093/iob/obz026

**Published:** 2019-10-25

**Authors:** N R Bressman, J W Love, T W King, C G Horne, M A Ashley-Ross

**Affiliations:** 1 Department of Biology, Wake Forest University, Winston-Salem, NC 27109, USA; 2 Maryland Department of Natural Resources, Annapolis, MD 21401, USA

## Abstract

Most fishes known for terrestrial locomotion are small and/or elongate. Northern snakeheads (*Channa argus*) are large, air-breathing piscivores anecdotally known for terrestrial behaviors. Our goals were to determine their environmental motivations for emersion, describe their terrestrial kinematics for fish 3.0–70.0 cm and compare kinematics among four substrates. For emersion experiments, *C. argus* was individually placed into aquatic containers with ramps extending through the surface of the water, and exposed to 15 ecologically-relevant environmental conditions. For kinematic experiments, fish were filmed moving on moist bench liner, grass, artificial turf, and a flat or tilted rubber boat deck. Videos were digitized for analysis in MATLAB and electromyography was used to measure muscular activity. Only the low pH (4.8), high salinity (30 ppt), and high dCO_2_ (10% seltzer solution) treatments elicited emersion responses. While extreme, these conditions do occur in some of their native Asian swamps. Northern snakeheads >4.5 cm used a unique form of axial-appendage-based terrestrial locomotion involving cyclic oscillations of the axial body, paired with near-simultaneous movements of both pectoral fins. Individuals ≤3.5 cm used tail-flip jumps to travel on land. Northern snakeheads also moved more quickly on complex, three-dimensional substrates (e.g., grass) than on smooth substrates (e.g., bench liner), and when moving downslope. Release of snakeheads onto land by humans or accidentally by predators may be more common than voluntary emersion, but because northern snakeheads can respire air, it may be necessary to factor in the ability to spread overland into the management of this invasive species.

## Synopsis

Auftauchen und Landbewegung des Argus-Schlangenkopffisches (*Channa argus*) auf mehreren Substraten (Emersion and terrestrial locomotion of the northern snakehead (*Channa argus*) on multiple substrates) Die meisten Fische welche für Landbewegung bekannt sind, sind klein und/oder länglich. Argus-Schlangenkopffische (*Channa argus*) sind große, luftatmende Fischfresser, die anekdotenhaft für ihr terrestrisches Verhalten bekannt sind. Unser Ziel war es, die umweltbedingten Motivationen für ihr Verlassen des Wassers zu bestimmen, die terrestrische Kinematik für Fische von 3, 0 bis 70, 0 cm zu beschreiben und die Kinematik zwischen vier Substraten zu vergleichen. Für Auftauch-Experimente wurden C. argus einzeln in Wasserbehälter gesetzt, die mit Rampen versehen waren, welche durch die Wasseroberfläche führten, und wurden 15 ökologisch relevanten Umweltbedingungen ausgesetzt. Für kinematische Experimente wurden Fische gefilmt, die sich auf einer feuchten Laborunterlage, Gras, Kunstrasen und einem flachen oder geneigten Gummibootsdeck bewegten. Videos wurden für die Analyse in MATLAB digitalisiert und Elektromyographie wurde verwendet, um die Muskelaktivität zu messen. Nur Treatments mit niedrigem pH (4, 8), hohem Salzgehalt (30 ppt) und hohem dCO_2_ (10%) lösten ein Verlassen des Wassers aus. Obwohl extrem, treten diese Bedingungen in einigen ihrer heimischen asiatischen Sümpfe auf. Argus-Schlangenkopffische > 4, 5 cm verwendeten eine einzigartige Form der terrestrischen Fortbewegung auf der Basis von Axialextremitäten, bei der zyklische Schwingungen des axialen Körpers mit nahezu gleichzeitigen Bewegungen beider Brustflossen einhergingen. Individuen ≤ 3, 5 cm benutzten „Schwanz-Flip-Sprünge“, um sich an Land zu fortzubewegen. Argus-Schlangenkopffische bewegten sich außerdem auf komplexen dreidimensionalen Substraten (z B. Gras) schneller als auf glatten Substraten (z. B. Laborunterlage) und wenn sie sich abwärts bewegten. Die Freisetzung von Schlangenkopffischen auf dem Land durch Menschen oder versehentlich durch Raubtiere ist möglicherweise häufiger als das freiwillige Verlassen des Wassers. Da Argus-Schlangenkopffische jedoch Luft atmen können, muss beim Management dieser invasiven Art möglicherweise ihre Fähigkeit berücksichtigt werden, sich über Land auszubreiten. Translated to German by F Klimm (frederike.klimm@biologie.uni-freiburg.de)

Emersión y locomoción terrestre de la cabeza de serpiente del norte (*Channa argus*) en múltiples sustratos (Emersion and terrestrial locomotion of the northern snakehead (*Channa argus*) on multiple substrates) La mayoría de los peces conocidos por locomoción terrestre son pequeños y/o alargados. Las cabezas de serpiente del norte (*Channa argus*) son grandes pesces piscívoros que respiran aire, anecdóticamente conocidos por sus comportamientos terrestres. Nuestros objetivos fueron determinar sus motivaciones ambientales para la emersión, describir su cinemática terrestre para peces de 3, 0 a 70, 0 cm y comparar la cinemática entre cuatro sustratos. Para los experimentos de emersión, *C. argus* se colocó individualmente en contenedores acuáticos con rampas que se extienden a través de la superficie del agua y fueron expuesto a quince condiciones ambientales ecológicamente pertinentes. Para los experimentos cinemáticos, los peces se filmaron moviéndose sobre un revestimiento de banco húmedo, césped, césped artificial y una cubierta de bote de goma plana o inclinada. Los videos se digitalizaron para su análisis en MATLAB y se usó electromiografía para medir la actividad muscular. Solo los tratamientos de bajo pH (4, 8), alta salinidad (30 partes por mil) y alto dCO_2_ (solución de agua de Seltz 10%) provocaron respuestas de emersión. Aunque son extremas, estas condiciones si ocurren en algunos de sus pantanos asiáticos nativos. Las cabezas de serpiente del norte >4, 5 cm usaron una forma única de locomoción terrestre basada en movimientos apéndiculares-axiales que involucra oscilaciones cíclicas del cuerpo axial, junto con movimientos casi simultáneos de ambas aletas pectorales. Los individuos de ≤3, 5 cm usaron saltos de cola para moverse en tierra. Las cabezas de serpiente del norte también se movían más rápidamente en sustratos tridimensionales complejos (ej., césped) que en sustratos lisos (ej., revestimiento de banco), y al moverse cuesta abajo. La liberación de cabezas de serpiente en la tierra por humanos o accidentalmente por depredadores puede ser más común que la emersión voluntaria, pero debido a que las cabezas de serpiente del norte pueden respirar aire, puede ser necesario tener en cuenta la capacidad de propagarse por tierra en el manejo de esta especie invasora. Translated to Spanish by YE Jimenez (yordano_jimenez@brown.edu)

## Introduction

A diversity of fishes exhibit a variety of terrestrial behaviors for a range of reasons, such as to escape onto land away from aquatic predators (Walker 1952; Goodyear 1970; Abel et al. 1987; Martin et al. 2004; Swanson and Gibb 2004; Blob et al. 2010; Hsieh 2010; Kawano and Blob 2013; Magellan 2015), to feed on land and access new resources (Mast 1915; Das 1928; Graham 1970; Wright and Raymond 1978; Graham et al. 1985; Gordon et al. 1985a; Van Wassenbergh et al. 2006; Taylor et al. 2008; Pronko et al. 2013; Van Wassenbergh 2013; Bressman et al. 2016, 2018a, 2018b), or to alleviate competition for resources (Huehner et al. 1985; Liem 1987). Environmental factors can also encourage amphibious fishes to emerge. Some species may leave the water if the dissolved oxygen (dO_2_) concentration is low (Ebeling et al. 1970; Graham 1970; Wright and Raymond 1978; Davenport and Woolmington 1981; Martin 1991; Sayer and Davenport 1991; Horn et al. 1999), pH is low (Davenport and Woolmington 1981; Robertson et al. 2015), temperatures are high (Ebeling et al. 1970; Gibson et al. 2015), hydrogen sulfide (H_2_S) concentrations are high (Abel et al. 1987), or dCO_2_ is high (Robertson et al. 2015). However, conditions that cause amphibious fishes to emerge can be species-specific (Ebeling et al. 1970; Davenport and Woolmington 1981; Abel et al. 1987; Liem 1987; Sayer and Davenport 1991; Wright and Turko 2016). In order to understand the mechanisms for overland movement by a species, it is important to investigate emersion on a species-by-species basis as broad generalizations are less informative. By developing a better understanding of factors that influence emersion, we may be able to improve management of invasive amphibious species, allowing us to predict when terrestrial behaviors and dispersion are most likely for each species.

Once on land, amphibious fishes use a variety of locomotor behaviors to move, with some relying on the axial body, some relying on appendages, and some relying on both. Appendage-based terrestrial locomotion is restricted to mudskippers (Oxudercinae) because they have highly modified pectoral fins with increased range of motion (Swanson and Gibb 2004; Pace and Gibb 2009, 2014; Kawano and Blob 2013). Axial-appendage-based terrestrial locomotion in fishes is more common (Johnels 1957; Pace and Gibb 2014; Standen et al. 2014, 2016), as a lower degree of morphological specialization is needed (Bressman et al. 2018a). Both appendage and axial-appendage-based terrestrial locomotion are limited to relatively small fish because it is difficult for large fish to support their body weight out of water on soft fin rays. An exception is walking catfish (*Clarias* spp.), which can reach lengths up to 40 cm and “walk” on land, supporting their weight on stiffened pectoral spines during axial-appendage-based locomotion (Johnels 1957; Pace and Gibb 2014). Larger amphibious fishes, such as eels (Anguilliformes; Gillis 1998; Gillis 2000; Gillis and Blob 2001), ropefish (*Erpetoichthys calabaricus*; Pace and Gibb 2011), and African lungfish (*Protopterus annectens*; Horner and Jayne 2014; Falkingham and Horner 2016) are typically very elongate and use axial locomotion while on land. With the exception of lungfish, which use their heads as a pivot while they thrust forward with their axial body (Horner and Jayne 2014; Falkingham and Horner 2016), these elongate fishes are able to use aquatic anguilliform locomotion on land, seemingly slithering like snakes using lateral undulation (Jayne 1986).

Previous studies of terrestrial locomotion in fish have mostly measured performance on relatively smooth, simple substrates, such as glass or damp bench liner (Pace and Gibb 2009; Gibb et al. 2011; Bressman et al. 2013, 2016, 2018a; Kawano and Blob 2013). However, substrate can impact terrestrial performance. Standen et al. (2016) found that the Senegal bichir (*Polypterus senegalus*), which use axial-appendage-based terrestrial locomotion, changes their gaits on more complex substrates. Hawaiian waterfall-climbing gobiids also improve performance on some rougher substrates by gaining better purchase (Blob et al. 2006). Studies of fish terrestrial locomotion on smooth substrates may underestimate their capabilities in natural settings, so it is important to investigate fish terrestrial performance on a variety of substrates.

Snakeheads (Channidae) are large, piscivorous fishes that can respire air using an accessory suprabranchial organ (Das 1928; Glass et al. 1986; Liem 1987; Lee and Ng 1994; Chew et al. 2003; Courtenay and Williams 2004; Li et al. 2017). Snakeheads of the genus *Channa*, which are native to warm temperate waters of southeastern Asia, are capable of reaching 1.8 m and 30 kg and are tolerant of very high acidities and extreme anoxic conditions (Das 1928; Lee and Ng 1994; Courtenay and Williams 2004; Li et al. 2017). Some species can survive for at least 20 h out of water and are known for terrestrial behaviors (Das 1928). Snakehead terrestrial locomotion has been described variously as a “rowing” of the pectoral fins (Das 1928), a “slither” (Pace and Gibb 2014), and a sinuous “crawl” or “wriggle” (Courtenay and Williams 2004). Essentially, snakeheads are known to move overland, but accurate and precise descriptions of their terrestrial behaviors and kinematics have been unavailable.

The prevalence of snakehead fishes in the hobby aquarium industry and their value for food in Asian markets have led to their invasion throughout the United States and Europe (Courtenay and Williams 2004; Orrell and Weigt 2005; Odenkirk and Owens 2007; Love and Newhard 2012, 2018). Currently, several different species have established spawning populations in the United States, including the northern snakehead (*Channa argus*), which are particularly prevalent in the Chesapeake Bay Watershed. Northern snakeheads are considered a particularly harmful and high-risk invasive species because they are very tolerant of extreme conditions and are piscivorous, which could adversely affect ecosystems and recreational fisheries (Courtenay and Williams 2004; Love and Newhard 2012).

The goals of this study are to determine environmental and ecological conditions that cause northern snakeheads to emerge from the water onto land, to provide a detailed description of the terrestrial locomotion of northern snakeheads, and to quantify their terrestrial kinematics. We investigated a wide range of factors that may elicit emersion in northern snakeheads, including many that elicit emersion in other amphibious species (Table 1; Sayer and Davenport 1991). Because substrate can impact terrestrial performance in fishes (Blob et al. 2006; Standen et al. 2016), we described and compared northern snakehead performance on multiple substrates, including natural, heterogenous substrates and artificial, homogenous substrates with different degrees of complexity. We hypothesized that substrate would impact kinematics and performance, with increased velocity on more three-dimensionally complex substrates, similar to *P. senegalus* (Standen et al. 2016). Additionally, because northern snakeheads grow relatively large for an amphibious fish, we sought to determine the effects of scaling on terrestrial kinematics and performance over a wide size range. As young northern snakeheads seem to resemble their adult forms morphologically (though their coloration changes), we hypothesized that scaling would minimally impact kinematics, and more greatly impact performance (i.e., velocity), as fish tend to move faster across ontogeny (Herrel and Gibb 2005). As one of the largest fishes known for moving on land, developing a better understanding of their terrestrial behaviors may help us further understand the scaling of locomotion in large vertebrates transitioning from water to land. Furthermore, as an invasive species that can potentially spread overland, understanding the environmental factors that influence their emersion can help identify situations in which dispersion may be most likely.

**Table 1:** *Channa argus* emersion treatments, results, and statistics

Treatment	Conditions	Rationale for treatment	Duration (min)	N	# Emerged	*P*-value	*Z*-value
Control	pH = 7.5, Temp = 20°C, [dO_2_] = 7.9 mg/L	Served as a comparison for the hypoxia treatment	60	11	0	—	—
Hypoxia	pH = 7.5, Temp = 19°C, [dO_2_] = 1.0 mg/L	Hypoxia elicits emersion in many amphibious species (Ebeling et al. 1970; Graham 1970; Davenport and Woolmington 1981; Sayer and Davenport 1991)	120	24	0	1	0
Darkness	Opaque tent blocking light	*Channallabes apus* emerge more frequently at night (Van Wassenbergh 2013)	60	12	0	1	0
Low pH	pH = 4.8	*Kryptolebias marmoratus* emerge under acidic conditions (Robertson et al. 2015)	60	12	5	0.008*	3.4
High pH	pH = 9.8	Deviations in pH from the neutral range found in natural waters have been found to elicit emersion in other killifish (Robertson et al. 2015)	60	12	0	1	0
Saltwater	30 ppt	The saltwater *P. novaeguineaensis* emerges when salinity is low (Gordon et al. 1985b) so the freshwater *C. argus* may emerge when salinity is high	60	12	3	0.039*	3.0
Brackish water	15 ppt	See Saltwater treatment above	60	12	0	1	0
Moderate dCO_2_	5% seltzer, pH= 6.5	*Kryptolebias marmoratus* emerge when dCO_2_ is high (Robertson et al. 2015)	60	12	0	1	0
High dCO_2_	10% seltzer, pH = 6.0	See Moderate dCO_2_ treatment above	45	12	6	0.002*	3.8
Low H_2_S	10 ppb	*Kryptolebias marmoratus* emersion rates increase as H_2_S concentrations increase (Abel et al. 1987)	60	12	0	1	0
Medium H_2_S	25 ppb	See low H_2_S treatment above	60	12	0	1	0
High H_2_S	350 ppb	See low H_2_S treatment above	60	12	0	1	0
Precipitation	Natural rain supplemented with artificial rain	*C. batrachus* emerge more frequently during precipitation (Liem 1987)	60	12	0	1	0
Crowding	All individuals in one container	*Anabas testudineus* and *C. batrachus* emerge more frequently under crowded conditions when competition is high (Liem 1987)	60	100	0	1	0
High Temperatures	19°C–40°C, increased by 0.5 C/min)	High water temperatures elicit emersion in *S. sanguineus* (Ebeling et al. 1970) and *K. marmoratus* (Gibson et al. 2015)	40	24	0	1	0

For various treatments and water conditions, the number (*N*) of individuals emerging from treatment conditions (# emerged) was tallied for analyses using two-proportion *Z*-tests, with the control treatment as a comparison. Bonferroni-corrected *P*-values are included.

* Significance at the α = 0.05 level.

## Methods

### Animals

Northern snakeheads (*C.argus* Cantor 1842; *n* = 351) were collected with the Maryland Department of Natural Resources (MDDNR) by electrofishing in Maryland tributaries of the Potomac River and drainage ditches adjacent to these tributaries. Specimens ranged greatly in size for kinematic experiments (total length [TL] = 3–70 cm). Of the specimens, 300 individuals were collected from the same school of fry guarded by the same parents, indicating they are likely all siblings. These individuals varied little in size (TL = 3.0–3.5 cm) and were used for emersion and kinematic experiments. Additionally, 11 individuals were collected from a separate school of fry (TL = 5.0–10.0 cm) and were used for the control treatment in the emersion experiments. Experimental animals were size-matched and housed together in several large, aerated aquaria with ambient lighting at 19–20°C at the Cedarville State Forest MDDNR Field Office in Brandywine, MD. At the conclusion of the experiments, all individuals were either kept by the MDDNR for future experiments or euthanized using tricaine mesylate (MS-222), according to IACUC guidelines, and fixed in formalin. Some of the specimens (*n* = 21) were then donated to the Harvard University Museum of Comparative Biology as two lots (Ichthyology 172824 and 172825). All experiments and procedures were conducted in accordance with Wake Forest University Institutional Animal Care and Use Committee (WFU IACUC) protocol A16-173.

### Emersion experiments

We used environmental stimuli that have been shown to influence emersion in previous studies (Table 1), including dissolved gasses, pH, temperature, salinity, light levels, and precipitation. Individual northern snakeheads (TL = 3.0–10.0 cm) were placed into plastic shoeboxes filled with well water (pH = 7.5, dO_2_ = ∼1.0 mg/L, temperature = 19°C). While the well water supplying the facility was hypoxic, we deemed it to have little impact on their behavior as they are obligate air-breathers (Das 1928; Liem 1987; Lee and Ng 1994; Courtenay and Williams 2004; Li et al. 2017) that can meet their metabolic demands through air-breathing under hypoxic conditions (Glass et al. 1986; Li et al. 2017). Therefore, the well water was used for all treatments. We aerated one treatment to be a normoxic control, so that we could compare it to the hypoxic treatments. The shoeboxes had a moist, wooden ramp extending from the bottom of the container through the surface of the water at an average angle of 27° ± 5° (Supplementary Fig. S1). A small hole was drilled into the boxes at 2 cm from the top to prevent overflow and assure water level were consistent between boxes and treatments. The fish were exposed to the treatments for ∼10 min before recording began. The treatments were as follows: control (normoxic water, ambient light, ambient temperature), hypoxia, darkness achieved using a tent made out of opaque tarps, low pH (4.8) achieved using hydrochloric acid, high pH (9.8) achieved using sodium hydroxide, brackish water (15 ppt) achieved using Instant Ocean saltwater mix, saltwater (30 ppt), moderate dCO_2_ (5% seltzer solution, pH = 6.5) high dCO_2_ (10% seltzer solution, pH = 6.0), low H_2_S concentration (10 ppb), achieved by adding an aqueous H_2_S solution (0.4%) to the water, medium H_2_S concentration (25 ppb), high H_2_S concentration (350 ppb), precipitation using containers placed outside during a rainstorm, supplemented with a hose with a shower nozzle and a hole near the top of the containers to prevent overflowing, crowding of 100 individuals in one container under control conditions, high temperatures with 24 individuals in a shallow metal pan with a ramp, heated on a hot plate at a rate of 0.5°C/s from 19°C to 40°C. A step-wise method was chosen for this treatment because it prevented cooling of the water throughout the experimental duration. Rationales for each treatment are included in Table 1.

For each treatment, individuals were randomly selected without replacement from a large housing aquarium. Twelve individuals were selected for all treatments, except the control, hypoxia, high temperatures, and crowding treatments (*n* = 24, 24, 100, respectively). Aside from the hypoxia treatment that lasted 2 h and the high temperatures treatment that lasted 40 min, treatments lasted 1 h unless fish exhibited adverse effects, at which point the treatment ended. The high dCO_2_ treatment was terminated after 45 min because the fish had difficulty maintaining an upright position in the water column. Except for the precipitation treatment, all treatments were conducted indoors during the day (9 AM–5 PM), as northern snakeheads are diurnal (Courtenay and Williams 2004). All emersion experiments took place in July 2018, except the control treatment, which had a similar set-up to the other treatments and took place in July 2019. Responses to treatments were recorded with a GoPro Hero5 camera at 30 frames per second (fps) for behavioral observations, except for the darkness treatment, which we observed from inside of the tent using night vision goggles. To limit the effects of human presence, an observer watched a livestream of the fish on a smartphone at the far end of the room, ∼3 m away from the experimental set-up. If a fish emerged onto the ramp with its mouth completely out of the water and any part of the gill opening out of the water (Supplementary Fig. S2) for more than 3 s, it was recorded as an emersion event. Fish that emerged remained in the treatments until completion. While multiple emergences were observed for some individuals, fish with at least one emergence during the treatment were classified as emergers.

### Kinematic data collection and analysis

To describe their terrestrial behaviors, northern snakeheads (*n* = 60; TL = 3.0–70.0 cm) were placed on a variety of substrates: (1) heterogeneous natural grass; (2) stiff but short, homogenous artificial grass turf; (3) smooth rubber boat deck with small bumps for grip; and (4) moist bench liner—plastic-backed paper towel—on a hard surface (Table 2). Individuals were allowed to move freely for up to 30 min while being filmed 90° dorsally by a stationary GoPro Hero5 camera at 30 fps, positioned ∼1.5 m above the substrate. Kinematic experiments took place in August and November 2017. Individuals that did not move during filming were excluded from this study. Eleven individuals on the bench liner and two on the turf were also filmed laterally during terrestrial locomotion using a GoPro Hero5 camera at 30 fps. These videos were only used for qualitative descriptions to help determine if *C. argus* lifts their bodies above the substrate and if they exhibit roll along the long axis. Individuals <4 cm were recorded with a stationary iPhone 7 at ∼90° and 0.5 m above the substrate at 240 fps, as their behaviors occurred more quickly than the larger individuals. In addition to the five individuals recorded on the flatboat deck, eight individuals were filmed on the boat deck while the boat was tilted. During this treatment, the water was very calm and we kept the distribution of boat passengers consistent, allowing us to achieve a consistent tilt of ∼10° for each fish. One successful sequence of locomotion was analyzed for each individual on only one substrate (sampling without replacement).

**Table 2:** Summary of kinematic and performance data on multiple substrates

Substrate	Length (cm)	DR Snout	DR Tail	DR COM	Velocity (cm/s)	Intrinsic Velocity (TL/s)	CC	WA Snout	WA Tail	WA COM		SF (Hz)		N
Boat Deck	49	0.338	0.146	0.566	9.69	0.204	0.449	0.218	0.277		0.089		1.06	5
SE	3.82	0.063	0.035	0.07	1.55	0.038	0.027	0.047	0.025		0.012		0.225	—
Tilted boat deck	47.3	0.319	0.172	0.732	16	0.341	0.424	0.207	0.304		0.114		0.959	8
SE	0.957	0.029	0.036	0.032	1.95	0.043	0.065	0.012	0.015		0.011		0.155	—
Bench liner	24.2	0.101	0.08	0.425	2.8	0.226	0.264	0.27	0.329		0.073		1.13	11
SE	7.08	0.013	0.014	0.049	0.517	0.051	0.037	0.029	0.006		0.009		0.135	—
Turf	48	0.172	0.122	0.446	7.81	0.207	0.273	0.28	0.323		0.084		0.735	14
SE	4.57	0.018	0.022	0.03	1.05	0.054	0.024	0.01	0.016		0.009		0.048	—
Grass forward crawl	61.8	0.178	0.145	0.541	16.1	0.269	0.337	0.205	0.236		0.068		1.39	6
SE	3.68	0.022	0.046	0.03	1.52	0.039	0.027	0.014	0.016		0.005		0.19	—
Backward crawl	61.96	0.055	0.021	0.146	3.8	0.065	0.102	0.223	0.277		0.064		0.883	2
SE	7.04	0.013	0.003	0.065	1.61	0.034	0.045	0.007	0.082		0.018		0.16	—

Substrate complexity was quantified using the chain-and-tape method for measuring rugosity (Risk 1972). This method involves placing a chain or string of known length over a three-dimensionally complex substrate, and measuring the distance between the endpoints. The ratio between the known length and measured length is used as a rugosity (R) index. Surface complexity was greatest on grass (*R* = 1.130), followed by the boat deck (*R* = 1.026), turf (*R* = 1.018), and bench liner (*R* = 1.012).

Observations from the terrestrial locomotion videos were used to qualitatively describe the terrestrial behaviors of northern snakeheads. To quantify the kinematics of locomotor behaviors (forward and backward crawling) from the videos, we used similar methods as Bressman et al. (2018a). Three points were manually tracked in the dorsal view using the DLTdv5 application of Dr Ty Hedrick’s Digitizing Tools (Hedrick 2008) in MATLAB: (1) the tip of the snout (head), (2) the tip of the tail (tail), and (3) the center of mass (COM). The COM was approximated as the anterior insertion of the dorsal fin using balance tests and preserved specimens, similarly to Bressman et al. (2018a). For these three points measured, displacement versus time was plotted to determine movement of various regions of each fish. Using these data and length measurements in ImageJ (Schindelin et al. 2012), wave amplitudes (WAs), curvature coefficients (CCs), velocities, stride frequencies (SFs), and distance ratios (DRs) were calculated for each stride. We define a single stride similarly to Bressman et al. (2018a): the lateral movement of the tail from the maximum curvature (when CC is smallest; see below) to the maximum curvature again on the same side of the body. If multiple, full strides were recorded for an individual, then the kinematic measurements for each stride in the sequence were averaged together. We define WA as the maximum amplitude of the head, tail, and COM as a percentage of TL and use it as a measure of how far laterally the snout, tail, and COM move during strides. A modified definition of CC from Brainerd and Patek (1998) and Bressman et al. (2018a) was used: a ratio of the distance between the tip of the snout and the tip of the tail, when the tail is maximally laterally displaced, to TL. Values of CC range between 0 and 1; a smaller value indicates greater curvature. We used CC to quantify lateral flexion. Velocity was calculated by measuring the distance between in-phase start and end points (i.e., when the head is at its first maximum amplitude to the left to when the head is at its last maximum to the left) of locomotor sequences, and dividing by duration of the sequence. To calculate SF, we counted the number of strides in a sequence and divided by duration of the sequence. The DR is a measure of how far laterally the head, tail, and COM move relative to the overall anterior movement, and is defined as the net displacement divided by the gross displacement (Pace and Gibb 2014; Bressman et al. 2018a). The DR served as a proxy for the linearity of the path traveled, providing a measure of how effective each locomotor strategy was for producing forward movement in the species compared to lateral movement.

### Electromyography

**Fig. 1:**
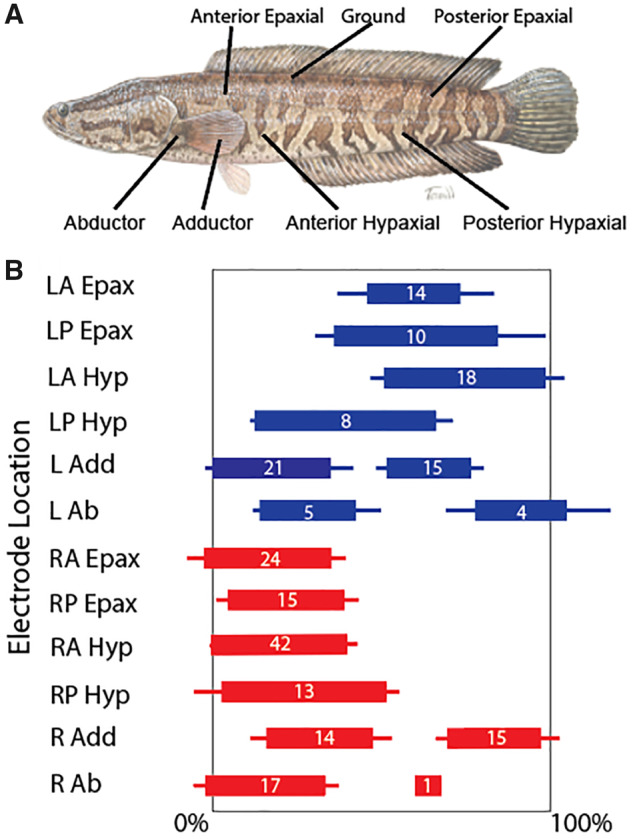
Muscle activity pattern during the terrestrial forward crawl of northern snakeheads. (**A**) Each northern snakehead (*n* = 7) in the EMG experiments had six intramuscular fine-wire electrodes implanted on each side (blue = left, red = right) in the labeled muscles, as well as a ground electrode near the base of the dorsal fin (snakehead image by Susan Trammell). (**B**) Since their forward crawl is a cyclical behavior, the muscle activity pattern is shown from 0% to 100% for each stride, with 100% being 0% of the subsequent stride. The thick bars indicate the average start and end times of muscle activation, while the thin bars reflect the standard error (SE). The numbers on the thick bars show the number of strides recorded for each muscle, with two numbers present for the abductors and adductors because these muscles would activate up to two times per stride. Overall, there is a left–right activation pattern of axial muscles, while both pectoral fins are coordinated for each half of a stride. The RA Hyp was chosen as the starting frame of reference as it had the most strides recorded of any muscle. LA Epax, left anterior epaxials; LP Epax, left posterior epaxials; LA Hyp, left anterior hypaxials; LP Hyp, left posterior hypaxials; L Add, left adductor; L Ab, left abductor; RA Epax, right anterior epaxials; RP Epax, right posterior epaxials; RA Hyp, right anterior hypaxials; RP Hyp, right posterior hypaxials; R Add, right adductor; R Ab, right abductor.

Seven of the individuals that were filmed on the artificial turf were also simultaneously used for electromyography (EMG) in November 2017. Because northern snakeheads survive very well out of the water for extended periods of time (Das 1928) and would stay very still when implanted with electrodes, electrode implantation was performed quickly without anesthesia to avoid affecting their locomotor behaviors. Thirteen fine-wire electrodes were implanted intramuscularly into each of these snakeheads using 30 G syringe needles. The electrodes were implanted into the left and right pectoral fin abductors, pectoral fin adductors, anterior hypaxials, posterior hypaxials, anterior epaxials, and posterior epaxials, as well as a ground electrode implanted into the midline connective tissue near the base of the dorsal fin (Fig. 1A). Electromyograms of these muscles were recorded using AcqKnowledge software (BIOPAC Systems 2007). To synchronize the videos with the electromyograms, an LED was also connected to the AcqKnowledge software, which was manually flashed at the onset of locomotor behaviors. Due to limitations in sampling rates, only six muscles at a time (chosen at random) were recorded during each locomotor sequence, at a sampling rate of 2392.91 samples per second. Northern snakeheads were allowed to move freely until there was no slack in the wires, at which point we interfered to prevent them from dislodging the electrodes. Multiple sequences, in which different combinations of muscles were recorded, were captured for each individual. Using the EMG recordings from all individuals, the relative duration and start and end times of activation of all muscles relative to the kinematic events of the stride were patched together to determine the average muscle activity pattern of their forward locomotory behavior (Fig. 1B).

### Statistical analysis

Two-proportion *Z*-tests were performed on a TI-84 calculator to compare the proportion of individuals that emerged in each treatment to the control treatment. The *P*-values from these tests were adjusted for multiple comparisons using the Bonferroni correction in the p.adjust command in R (Wright 1992; R Core Team 2017).

Statistical analyses of kinematic data were performed in R using the standard statistics package (R Core Team 2017) and the jmv “jamovi” analysis package (The jamovi project 2019). Multiple analyses of covariance (MANCOVA) tests were used to determine if substrate type and TL affected kinematic parameters, and if there were interactions between the effects of size and substrate. Because of unequal sample sizes and failure to meet the assumption of homogeneity of variance, ranked MANCOVAs were used in place of parametric tests. A significant effect of substrate type on the response variables was determined using the Pillai’s Trace test statistic. Independent Kruskal–Wallis rank sum tests were used to determine how dependent kinematic variables differed among substrate types (independent variable). Spearman’s rank-order tests and individual linear regressions were used to determine if TL predicted dependent kinematic variables. Welch’s two sample *t*-tests were used to compare kinematic parameters between forward and backward crawling behaviors, as well as between individuals on turf with and without electrodes implanted.

## Results

### Emersion responses

In both the control and hypoxia treatments, northern snakeheads showed no signs of distress, which could have included erratic swimming, inability to remain horizontal in the water column, and rapid ventilation. The fish in the darkness, high pH, brackish, low H_2_S, medium H_2_S, precipitation, and crowding treatments appeared to behave normally as well. While we did not quantify swimming movements or air gulps, in the high dCO_2_, low pH, and saltwater treatments, the fish did exhibit signs of distress, appearing to swim more frequently and quickly than in the hypoxia treatment. In the moderate dCO_2_ treatment, the fish noticeably swam less frequently than in the control treatment, but maintained an upright position. The fish in the higher temperature treatment were more active and gulped air from the surface more frequently as temperature increased.

No northern snakeheads emerged from the water during the control or hypoxia treatments (Table 1), but individuals did emerge when exposed to acidic conditions, high salinity, and high levels of dCO_2_ (Supplementary Fig. S2). The first emersions in these treatments were at 15, 40, and 10 min, respectively. Emersion events lasted from 2 s to 20 min. Typically, the individuals that emerged would alternate emersion and immersion, switching periodically after the initial emersion. In the rest of the treatments, the fish did not emerge, nor did they behave differently than the control or hypoxia treatments.

### Kinematics

**Fig. 2:**
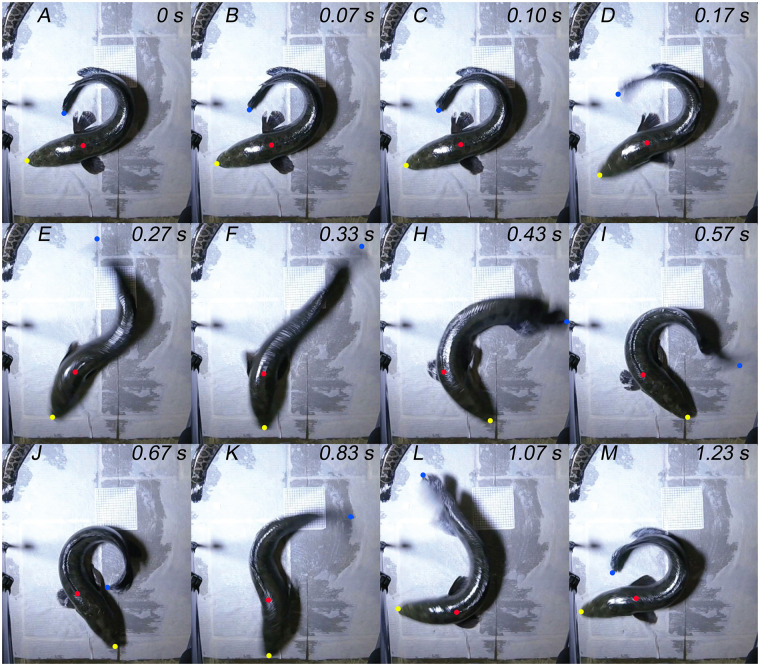
Northern snakehead forward crawl behavior. (**A**) A northern snakehead begins crawling by (**B**) moving its head toward the left and swinging the tip of its tail laterally toward the right. (**C**) The head and tail continue to move as the left pectoral fin begins retracting. (**D**) The right pectoral fin begins to retract, and the tail tip has achieved its approximate maximum lateral amplitude as it begins swinging toward the body’s midline. (**E**) Both pectoral fins are fully retracted as the head crosses the midline of the body. The fish rolls along its longitudinal axis partially onto its left pectoral fin, while the posterior axial body appears to push against the substrate. (**F**) The pectoral fins begin to protract as the tail crosses the body’s midline. (**H**) The fish rolls along its longitudinal axis toward the right side of its body as the tail tip rapidly increases in amplitude toward the left. (**I**) Both pectoral fins are fully protracted and the head has reached its maximum amplitude on the left side of the body. (**J**) The head begins moving back toward the right as the tail tip has reached its maximum amplitude on the left. The pectoral fins begin to retract. (**K**) The head and tail are both moving toward the right side of the body as both pectoral fins are fully retracted. The fish rolls partially onto its right pectoral fin, as the posterior axial body appears to push against the substrate. (**L**) The head has approximately reached its maximum amplitude as the fish rolls toward the left side of its body and rapidly increases the tail tip amplitude on the right side of the body. (**M**) The tail tip has reached its maximum amplitude on the right side of the body and the head begins moving back toward the left side of the body, starting a new stride. The three points tracked—tip of the snout, anterior insertion of the dorsal fin/COM, tip of the tail—are shown by yellow, red, and blue dots, respectively.

While on land, northern snakeheads >4.5 cm in TL adopted an upright posture, regardless of substrate. To move anteriorly while upright on a terrestrial substrate, northern snakeheads used coordinated cycles of pectoral fin and axial body movements (Fig. 2; Supplementary Video S1). This crawl-like behavior is a form of axial-appendage-based terrestrial locomotion and had an average stride duration of 1.162 s ± 0.411 s. Crawls lasted an average of 3.55 s ± 2.53 s per sequence, but new sequences often began as soon as 1 s after the previous, suggesting northern snakeheads move in short bursts. Coordination between the pectoral fins was variable. In some instances, both pectoral fins appeared to retract almost simultaneously as the tail and head began to swing toward each other from one side of the body to the other during each half of a stride, repeating on the other side of the body to complete a stride (Fig. 2; Supplementary Video S1). In other instances, the pectoral fins were out-of-phase, with one retracting as the other protracts (Supplementary Video S2). Additionally, within the same sequence, some fish had their fins in-phase for some strides and out-of-phase for others (Supplementary Video S3). The pectoral fins maintained contact with the substrate as they retracted, but lifted off the substrate as they protracted (Supplementary Video S4). Meanwhile, the axial body oscillated while maintaining contact with the substrate, but the fish rolled slightly along their longitudinal axis onto the pectoral fin of the side that their head and tail moved toward (Fig. 2; Supplementary Video S1). During this rolling, their posterior axial body appears to push against the substrate to provide forward thrust. In addition to potentially providing thrust, the pectoral fins help support the fish, likely keeping them upright and preventing excessive rolling. The larger fish that were filmed laterally (>30 cm) did not lift their ventral surface above the substrate (Supplementary Video S3).

**Fig. 3:**
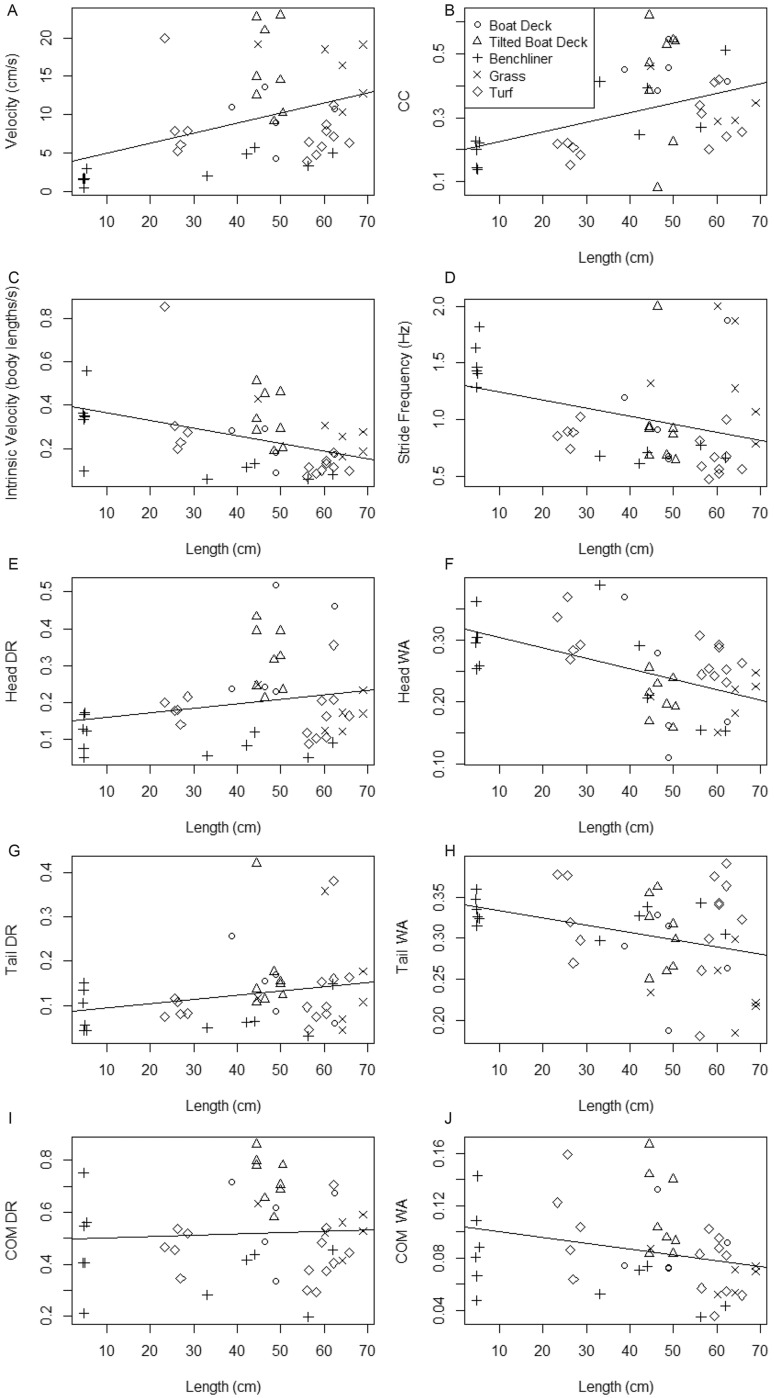
Effects of substrate and length on kinematics and performance. Kinematic and performance parameters of the snakehead forward crawling behavior on each substrate (symbols in panel B) are plotted on the *y*-axes against length. The regression line includes all substrates, to show the overall effect of length. CC = Curvature Coefficient, DR = Distance Ratio, WA = Wave Amplitude, COM = Center of Mass.

**Fig. 4:**
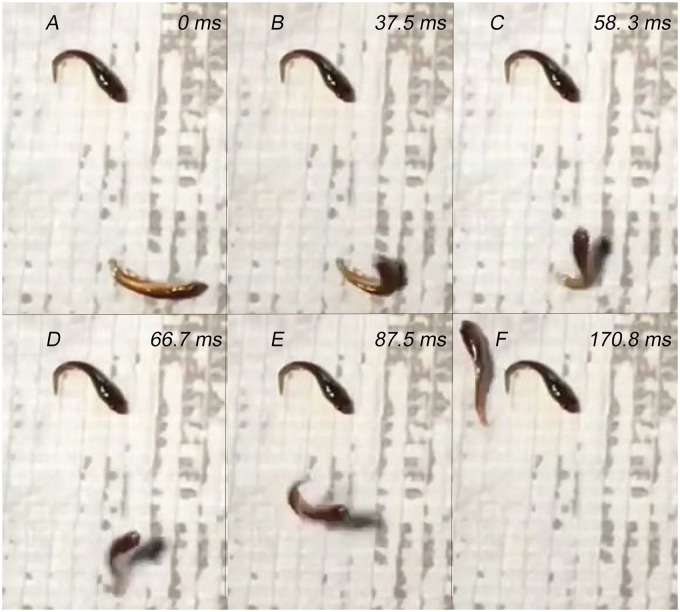
Juvenile northern snakehead tail-flip jump. (**A**) A juvenile northern snakehead (bottom right of the panel, ∼3.2 cm in TL) begins a tail-flip by lying on its lateral surface, and then (**B**) lifts its head above the substrate and bends it towards the tail, making a C-shape with the body. (**C**) Once the fish is maximally bent, it rapidly pushes against the substrate with its caudal peduncle, (**D**) launching itself into a ballistic flight path. (**E**) While the fish is airborne, it can travel several body lengths before landing (**F**) next to a stationary northern snakehead.

In addition to the forward crawling behavior, two individuals exhibited a backward crawling behavior on grass. This behavior involved axial movements with pectoral fins protracted farther than during forward crawling (up to 180°), with limited retraction (Supplementary Video S5). The axial body used a less oscillatory and more undulatory motion for backward crawling than forward crawling, and initiated the behavior at the tail. However, this behavior was rarer, only observed twice out of the 46 crawling sequences recorded, and had significantly lower head and tail DRs, absolute velocity, and relative velocity (velocity scaled to body length; Tables 2 and 3; Fig. 3). Northern snakeheads <3.5 cm (*n* = 18), which still displayed juvenile coloration, did not use an axial-appendage-based crawling behavior for terrestrial locomotion, but used tail-flipping behavior when out of the water (Fig. 4; Supplementary Video S7). These individuals were excluded from the quantitative kinematic analysis, as they perform a different behavior than crawling. No individuals >3.5 cm were observed to use tail flip jumps, and no individuals <4.5 cm were observed using an effective axial-appendage-based crawling behavior, so the transition from tail-flipping to crawling occurs between 3.5 and 4.5 cm in TL. A few smaller individuals (3.0–3.5 cm in TL) were observed attempting crawl-like behaviors, but were unable to achieve full strides or net displacement.

**Table 3:** Summary of statistical tests comparing effects of substrate and size on response variables

Test	Test statistics	DR snout	DR tail	DR COM	Velocity (cm/s)	Relative velocity (TL/s)	CC	WA snout	WA tail	WA COM	SF (Hz)
Kruskal–Wallis	*P*	<0.001*	0.039*	<0.001*	<0.001*	0.341	0.012*	0.007*	0.007*	0.026*	0.024*
	χ^2^	28.3	10.1	19.7	24.4	2.76	12.9	14.0	14.0	11.1	11.279
	df	4	4	4	3	3	4	4	4	4	4
Spearman’s	*P*	0.461	0.199	0.924	<0.001*	0.011*	0.011*	<0.001*	0.056	0.028*	0.053
	*r*	0.114	0.197	0.015	0.560	−0.417	0.381	−0.516	−0.290	−0.332	−0.294
	*S*	12,570	11,387	13,979	3421	11,008	8778	21,507	18,301	18,897	18,357
Boat deck *t*-test	*P*	0.799	0.607	0.076	0.028*	0.037*	0.728	0.834	0.382	0.151	0.711
	*t*	0.267	0.530	2.17	2.53	2.38	0.359	0.223	0.932	1.55	0.384
	df	5.79	10.5	5.74	11.0	10.7	9.17	4.53	6.99	10.1	7.69
Turf *t*-test	*P*	0.956	0.943	0.946	0.299	0.448	0.812	0.944	0.600	0.237	0.879
	*t*	0.057	0.074	0.070	1.13	0.804	0.244	0.734	0.542	1.26	0.155
	df	8.28	7.23	8.80	6.43	6.77	11.2	7.74	9.65	10.4	11.9

Kruskal–Wallis tested for an effect of substrate on response variables, and Spearman’s rank-order correlation tests tested for an effect of size on the other parameters, but backward crawling is excluded from these analyses. The boat deck *t*-test data refer to comparisons between parameters for flat and tilted boat decks, while the Turf *t*-test data refer to comparisons between parameters for snakeheads moving on turf with and without electrodes implanted.

* Significance at the α = 0.05 level.

### EMG

The EMG analysis revealed an alternating left–right pattern of muscle activation in northern snakeheads during their forward crawling behavior on turf (Supplementary Video S6), particularly in the axial muscles (Fig. 1B;Supplementary Fig. S3). There appeared to be only a slight delay in activation between the anterior and posterior epaxial and hypaxial muscles—which is particularly evident on the right side, for which there was a greater sample size. Thus, the axial movements of northern snakeheads during terrestrial crawling are closer to a standing than traveling wave, aligning with typical axial-appendage-based locomotion characteristics. There was a much larger phase shift, however, for ipsilateral pectoral fin abductor and adductor activation. Furthermore, both the abductors and adductors often activated twice per stride, but not always (Fig. 1B), as indicated by the unequal number of recordings between the first and second muscle activations in a stride. The EMG data show the abductors on one side synchronized with the contralateral adductors in a paddling motion; the fish retracted one pectoral fin as the contralateral pectoral fin protracted (Supplementary Video S2), and the contralateral axial muscles began contracting. Then, the first pectoral fin protracted as the contralateral pectoral fin retracted and the contralateral axial muscles completed contraction.

### Effects of substrate

Terrestrial performance and kinematics of northern snakeheads differed significantly across substrates (*P* < 0.001, *F* = 4.22, df1 = 40.0, df2 = 128) after accounting for a significant effect of TL (*P* < 0.001, *F* = 4.96, df1 = 10.0, df2 = 29; Tables 2 and 3; Fig. 3). Tilting the boat deck had no significant effects on kinematics, but did increase absolute velocity and relative velocity (Tables 2 and 3; Fig. 3); therefore, the tilted boat deck data were included in neither the ANOVAs nor correlation tests for velocity and relative velocity. While most individuals moved downslope on the tilted boat deck, one individual moved upslope, but was not included in the statistical analysis. Electrode implantation had no significant effects on any kinematic or performance parameters for individuals moving on turf (Table 3), so data from individuals with electrodes implanted were combined with the rest of the turf data for comparisons to other substrates. Overall, small northern snakeheads moved differently than large ones. As length increased, absolute velocity and CC significantly increased, while relative velocity and head and COM WA significantly decreased (Tables 2 and 3; Fig. 3).

Northern snakeheads performed differently on different substrates, moving most quickly on grass (Table 2; Fig. 3). Substrate type had significant impacts on head, tail, and COM DRs, velocity, CC, SF, and head, tail, and COM WAs (Table 3). Northern snakeheads had greater DRs on more complex substrates like grass and the boat deck. This is further supported by the greatest velocities on grass (Supplementary Video S8) and the boat deck (Supplementary Video S2), and the lowest on bench liner (Table 2; Supplementary Video S1). The CCs were greatest (meaning curvature was least) and WAs were least on the boat deck and grass. Additionally, grass produced the highest SF (Table 2; Fig. 3).

## Discussion

### Emersion and behaviors

These are the first documented cases of environmentally-motivated emersion in the northern snakehead. Of the tested emersion treatments, only high acidity, high dCO_2_, and high salinity elicited emersion responses in some, but not all, individuals. Future experiments could investigate intraspecific variation that leads only some individuals to emerge under these conditions. Snakeheads may naturally emerge under different, less extreme conditions, but these may be rare occurrences that would be difficult to observe by random chance. High dCO_2_ negatively affects fish by causing cardiac arrest, respiratory acidosis, hyperventilation, and increased cortisol levels (Crocker and Cech 1998; Ishimatsu et al. 2004). Environmental hypercapnia can occur in still waters with decaying material, such as in drainage ditches where some of the northern snakeheads were collected. Individuals from those ditches lived in a few inches of muddy, stagnant water. It is possible that they may emerge when the dCO_2_ reaches a certain threshold, or the water dries up, isolating the fish in small, unconnected puddles. Emersion would be a means of escaping the poor quality and limited resources of the isolated puddles. High dCO_2_ also elicits emersion responses in the distantly-related mangrove rivulus (*Kryptolebias marmoratus*; Cyprinodontiformes; Robertson et al. 2015), suggesting this may be a factor that encourages emersion in many amphibious fishes.

Northern snakeheads emerged under acidic conditions, as does *K. marmoratus* (pH 5.5; Robertson et al. 2015). Acidic conditions can be harmful to fish, as it can disrupt ionoregulation and acid–base balance (Schofield 1976), so emersion may be a means to avoid these negative effects. Basic conditions (pH 9.8) did not elicit emergence. Many *Channa* spp. are tolerant to extremely high acidity and high basicity, live in waters with a pH as low as 2.80 and as high as 9.6 (Lee and Ng 1994), so our acidic treatment was within their natural pH tolerance. However, their emersion response to high acidity suggests that in the acidic blackwaters of east and southeast Asia where many *Channa* species are native (Lee and Ng 1994), terrestrial emersion may be a common occurrence in snakehead species. While our basic treatment may have had a pH greater than in much of the native range of *Channa*, the pH in parts of the Potomac River system where northern snakeheads are invasive can be greater than the pH we used, sometimes exceeding 10.0 (Maryland Department of Natural Resources 2018). Even still, northern snakeheads did not seem to be perturbed by the basic conditions, with no individuals emerging, suggesting they are more sensitive to acidic conditions than basic.

The brackish salinity treatment (15 ppt) elicited no emersion response, but northern snakeheads are somewhat tolerant of brackish conditions, with an upper limit of 18 ppt (Li et al. 2008; Snakehead Plan Development Committee, submitted for publication). However, the higher salinity treatment (30 ppt) exceeded their upper salinity tolerance and elicited emersion. As a freshwater fish, northern snakeheads would have difficulty osmoregulating in saltwater (Evans 2008). They may be more likely to emerge in tidal regions, such as in the tidewaters of the lower Potomac River system where they are invasive (Orrell and Weigt 2005; Odenkirk and Owens 2007; Love and Newhard 2012; 2018), or in coastal regions prone to flooding that may have rapid influxes of saltwater. However, their emersion demonstrates an intolerance of saltwater, making their expansion via coastal lagoons or embayments unlikely. While no other amphibious species has yet been documented to emerge due to increased salinity, saltwater mudskippers (*Periophthalmus novaeguineaensis*; gobiiformes) will emerge to avoid freshwater (Gordon et al. 1985b; Sayer and Davenport 1991). This suggests that water salinity may be a factor in terrestrial emersion of other amphibious fishes, particularly of species that inhabit variable tide pools and coastal freshwaters prone to flooding from the ocean.

In addition to the moderate dCO_2_, basic, and brackish treatments, no emersions were observed in the control, hypoxia, high temperature, H_2_S, darkness, crowding, and precipitation treatments. While hypoxia elicits emersion in many amphibious fishes that breathe air cutaneously (Ebeling et al. 1970; Graham 1970; Wright and Raymond 1978; Davenport and Woolmington 1981; Martin 1991; Sayer and Davenport 1991; Horn et al. 1999; Livingston et al. 2018), the hypoxic conditions of the well water (∼1 mg O_2_/L) did not elicit any emersion in northern snakeheads in the hypoxia treatment. The hypoxia treatment had no observable differences in behavior compared to the control treatment. While this level of hypoxia may be insufficient to induce emersion in all cutaneous-breathing amphibious fishes (Sloman et al. 2008; Mandic et al. 2009; Regan et al. 2011), snakeheads have an accessory air-breathing organ (Das 1928; Liem 1987; Courtenay and Williams 2004) that does not rely on dO_2_. Under low dO_2_ conditions, cutaneous-breathing amphibious fishes need to completely emerge from the water to extract oxygen from the air. However, northern snakeheads and other fishes that breathe air with swim bladders, lungs, or accessory air-breathing organ, such as tarpon (Megalopidae), gar (Lepisosteiformes), and lungfish (Dipnoi), can gulp air from the surface while staying submerged (Graham 1997). Furthermore, the aerial ventilation rates of northern snakeheads increase very little under hypoxic aquatic conditions (Glass et al. 1986), suggesting dO_2_ levels have little effect on these obligate air-breathers. However, seasonal physiological differences in another air-breathing fish (gulf killifish, *Fundulus grandis*) can impact its response to hypoxia (Love and Rees 2002), so northern snakeheads may also respond differently to hypoxia depending on the season. Nevertheless, respiring at the surface makes northern snakeheads more visible and vulnerable to aquatic, terrestrial, and aerial predators.

Some *Channa* spp., like *C. striata*, are tolerant to temperatures as high as 40°C (Lee and Ng 1994), so we likely covered the maximum extent of the thermal range of northern snakeheads without an emersion response. While emersion is used by Aplocheiloid killifishes and the Chilean clingfish (*Sicyases sanguineus*; gobiesociformes) during high water temperatures to cool down via evaporative cooling (Ebeling et al. 1970; Sayer and Davenport 1991; Robertson et al. 2015; Livingston et al. 2018), no such response was observed in northern snakeheads, similar to the shanny (*Blennius pholis*; blenniidae; Davenport and Woolmington 1981). Killifish and *S. sanguineus* lack accessory air-breathing organs, though, so it is possible that these fish may leave the water to avoid hypoxic aquatic conditions associated with higher temperatures, whereas northern snakeheads can continue breathing air from the surface in anoxic warm water.

We used an H_2_S concentration (350 ppb) that elicited a 100% emersion response in *K. marmoratus* (Abel et al. 1987), yet it had no effect on northern snakehead emersion. While high levels of H_2_S elicit emersion responses in other amphibious fishes, these environmental disturbances did not elicit emersion behaviors in juvenile northern snakeheads. They may not be as sensitive to H_2_S, or may just respond differently, with northern snakeheads becoming more lethargic at high H_2_S concentrations.

Neither darkness, crowding, nor precipitation elicited emersion responses. As visual predators with large eyes, northern snakeheads likely use vision to orient on land like other amphibious fishes, which respond to colors, shapes, reflectivity, and celestial objects for terrestrial orientation (Goodyear 1970; Aronson 1971; Bressman et al. 2016, 2018b). Therefore, it may be unlikely for them to emerge from the water at night, as supported by the lack of emersion in the darkness treatment. Additionally, northern snakehead fry typically live close together in large numbers in a bait ball-like manner. Crowded conditions are standard for the fry, which is perhaps why these conditions did not elicit emersion. Crowding may limit resources over longer periods of time and cause waste to accumulate, which could encourage emersion, but long-term studies on the effects of crowding on emersion are needed. The range expansion of northern snakeheads in the Chesapeake Bay Watershed appears to be correlated with rainfall and flooding (Love and Newhard 2018), yet our precipitation treatment did not elicit emersion. However, we isolated the effects of precipitation to only include surface perturbations by falling water for a relatively short period of time. It may be that northern snakeheads preferentially take advantage of higher water levels during substantial precipitation events to expand their range aquatically rather than terrestrially, as staying in the water shields them from exposure to land predators and potential desiccation.

While the conditions that caused emersion in our experiments are uncommon in their non-native range in the United States, highly acidic and hypercapnic conditions are more common in some of the native range of northern snakeheads and other snakehead species, particularly in Asian swamps and blackwater river systems (Lee and Ng 1994). Emersion may be a means to escape unfavorable conditions in these habitats, either until conditions improve or to find more suitable bodies of water. In the United States, accidental and intentional releases onto land by human fishermen and predators may be a more common than environmentally-motivated emersion, but can nonetheless facilitate overland movements.

### Biomechanics

Adult northern snakeheads can move effectively on land using a form of axial-appendage-based locomotion. While other amphibious fishes also use axial-appendage-based terrestrial locomotion, including walking catfishes (Das 1928; Johnels 1957; Pace and Gibb 2014; Pace 2017), bichirs (Standen et al. 2014, 2016), *Cryptotora thamicola* (Flammang et al. 2016), and the tide pool sculpin (*Oligocottus maculosus*; Bressman et al. 2018a), northern snakehead is the only species described to use both pectoral fins almost simultaneously, at least in some instances, during axial-appendage-based locomotion. Mudskippers (*Periophthalmus* spp.) do use both of their pectoral fins simultaneously in a crutching motion that lifts their body off the substrate (Swanson and Gibb 2004; Pace and Gibb 2009; Kawano and Blob 2013; Wicaksono et al. 2017); however, mudskipper crutching is solely appendage-based locomotion, which is likely unfeasible for adult snakeheads to achieve as they are too large to lift their bodies up and support them on their soft pectoral fins that lack stiffened fin rays.

While some fishes of similar length are capable of terrestrial locomotion, like the ropefish (*E.calabaricus*; Pace and Gibb 2011), African lungfish (*P.annectens*; Horner and Jayne 2014; Falkingham and Horner 2016), and American eel (*Anguilla rostrata*; Gillis 1998; Gillis 2000; Gillis and Blob 2001), these fish are more elongate and rely on axial-based terrestrial locomotion. Since it appears that adult northern snakehead is too large for appendage-based locomotion, and not elongate enough for axial-based locomotion, they exhibit a distinct, novel form of axial-appendage-based locomotion that allows them to move their large bodies. Most axial-appendage-based amphibious fishes lift their COM above the substrate (Johnels 1957; Pace and Gibb 2014; Standen et al. 2014, 2016; Bressman et al. 2018a). However, while we only tested a subset of substrates, our results suggest that northern snakehead terrestrial locomotion does not include a vertical component. Unlike *O. maculosus* and *C. batrachus*, which consistently alternate their pectoral fins (Johnels 1957; Pace and Gibb 2014; Bressman et al. 2018a), northern snakeheads have variable pectoral fin coordination, suggesting kinematic flexibility in their terrestrial locomotion. This variability in pectoral fin coordination, which may not have been fully captured by EMG due to a limited sample size, may aid in moving over uneven surfaces, such as grass. However, we consistently recorded large phase shifts in ipsilateral pectoral fin abductors and adductors. This is to be expected of antagonistic muscles in cyclical fish behaviors, such as during labriform swimming in labrids (Westneat 1996; Westneat and Walker 1997). The low ipsilateral pectoral muscle variation suggests that individual fin movements during terrestrial locomotion are stereotyped, but coordination between fins is variable, which may increase locomotor flexibility. Alternatively, achieving effective terrestrial locomotion with variable pectoral fin usage suggests that pectoral fins may be relatively unimportant for propulsion compared to the axial body, and instead may be used primarily for balance during terrestrial locomotion.

Like *O. maculosus*, which have very similar COM DR on bench liner (0.38; Table 2; Bressman et al. 2018b), northern snakeheads have flexible pectoral fin rays compared to *Clarias* spp., which have rigid pectoral spines. The COM DR of northern snakeheads and *O. maculosus* are about half that of *Clarias* spp. (∼0.70–0.80; Table 2; Pace and Gibb 2014), suggesting rigid pectoral fins greatly improve terrestrial performance in fish that use axial-appendage-based terrestrial locomotion. Additionally, patterns of higher head and tail WA and lower COM WA movements have also been observed in *Clarias* spp. (Pace and Gibb 2014) and *O. maculosus* (Bressman et al. 2018a), suggesting this is a common pattern among fish that use oscillatory axial-appendage-based terrestrial locomotion.

Northern snakeheads moved most quickly over grass, the most complex substrate tested. The increased complexity may allow northern snakeheads to increase purchase on this substrate, and therefore performance, similar to how Hawaiian climbing gobies (gobiidae) are able to improve purchase and performance on some rougher substrates (Blob et al. 2006). Furthermore, the increased three-dimensionality would improve the axial portion of their terrestrial locomotion by providing more structure to push against laterally with the body, similarly to *P. senegalus* on more complex substrates (Standen et al. 2016).

When the boat deck was tilted, northern snakeheads were able to move significantly faster, suggesting they may work with gravity to more effectively move down slopes, similarly to mosquitofish (*Gambusia affinis*; Boumis et al. 2014) and mangrove rivulus (*K.marmoratus*; Bressman et al. 2018b). As water tends to pool at the bottom of slopes, moving downslope could be beneficial, as it would improve a fish’s chances of finding water while on land. However, an individual did move up the tilted boat deck in a similar manner to the individuals on the flatboat deck. While accurate kinematic conclusions cannot be drawn from this one observation, it does show that northern snakeheads are capable of moving upslope and potentially out of bodies of water.

Unlike adults, northern snakehead fry use caudally-directed tail-flip jumps to locomote on land, like many killifishes (Cyprinodontiformes; Bressman et al. 2016, 2018b; Gibb et al. 2013; Gibb et al. 2011; Mast 1915; Minicozzi et al. 2019; Perlman and Ashley-Ross 2016; Pronko et al. 2013). The transition between terrestrial locomotor behaviors occurs between 3.5 and 4.5 cm in TL, which is also when they change from their orangey fry coloration to mottled green, more closely resembling adults. Modest ontogenetic shifts in kinematics are not rare (Drucker 1996; Domenici 2001; Gibb et al. 2005), but complete ontogenetic shifts in locomotor behaviors such as this have not been described in fishes. A drastic shift to a new locomotor behavior is typically the result of extreme metamorphoses, such as in amphibians and lepidopterans. Several cyprinodontiform species are able to tail-flip at TL up to 11 cm (Bressman et al. 2016; Minicozzi et al. 2019), so the transition to crawling in snakeheads larger than 4.5 cm may not be because of size limitations. There may be morphological changes occurring as the coloration changes that may reduce their ability to tail-flip. Crawling may also become more effective and efficient on land once northern snakeheads reach 3.5–4.5 cm, at which point they transition terrestrial locomotor behaviors. Their mass distribution may also be different than in mummichogs and other tail-flipping killifish, potentially limiting their ability to tail-flip at a smaller size.

In northern snakeheads larger than 4.5 cm, CC increases and WA decreases with body length, which could be associated with increased vertebral flexural stiffness through ontogeny of fishes (Gibb et al. 2005). As they grow and their vertebral columns stiffen, the maximum extent to which they can bend their axial bodies laterally may become reduced. However, the increase in vertebral flexural stiffness with ontogeny may allow for improved energy transfer between axial muscles and the axial skeleton (Gibb et al. 2005). This could partially explain the ontogenetic increase in northern snakeheads terrestrial velocity (Gibb et al. 2005), which is consistent with the ontogenetic increase in the aquatic velocities of vertebrates (Domenici 2001). The ontogenetic decrease in relative velocity in northern snakeheads is also consistent with other vertebrates (including frogs, salamanders, sharks, fish, and mammals; Curtin and Woledge 1988; Rome et al. 1990; Altringham et al. 1996; James et al. 1998; D′Aou^t and Aerts 1999; Hale 1999; Azizi and Landberg 2002; Gibb et al. 2005), as muscle shortening velocity decreases with increasing body size (Herrel and Gibb 2005). As in many other vertebrates, juvenile northern snakehead may have relatively high levels of locomotor performance compared to adults to improve escape responses when most vulnerable to predation (Herrel and Gibb 2005). We also observed a decreasing trend in SF as TL increased in northern snakeheads, consistent with previous observations of *O. maculosus* and *C. batrachus* (Johnels 1957; Pace and Gibb 2014; Bressman et al. 2018b). Scaling seems to affect SF not only within species of amphibious fishes, but between species, likely because muscle shortening velocity is inversely related to body size (Herrel and Gibb 2005) and large fish need to move their bodies absolutely farther to achieve the same relative distance as small fish.

While there is a phase shift between anterior and posterior axial muscles, this shift is relatively small given the distance between anterior and posterior electrodes. This suggests that northern snakehead axial movement during forward crawling behaviors is more oscillatory than undulatory on the oscillatory-undulatory spectrum, and is more similar to *P. annectens* (Horner and Jayne 2014) and *O. maculosus* (Bressman et al. 2018a) than *E. calabaricus* (Pace and Gibb 2011) and *A. rostrata* (Gillis 1998, 2000; Gillis and Blob 2001), which use highly undulatory terrestrial locomotor behaviors. While undulatory behaviors for terrestrial locomotion are more easily derived from anguilliform locomotion (Gillis 1998, 2000; Gillis and Blob 2001), as they require few changes to their locomotor pattern between media, oscillatory behaviors for terrestrial locomotion are more reminiscent of salamander locomotion (Ashley-Ross et al. 2009) and require a higher degree of deviation from swimming kinematics. Furthermore, except for labriform swimmers (Westneat 1996; Drucker and Jensen 1997; Westneat and Walker 1997), most fish do not use both pectoral fins simultaneously during sustained swimming, including northern snakeheads (NRB’s, personal observations). Therefore, it is unlikely their steady swimming muscle pattern, which they use for constant-speed cruising (Langerhans 2009), is the origin of their terrestrial crawling muscle pattern. Snakeheads may use one pectoral fin or both simultaneously along with their axial body for unsteady swimming, which includes aquatic fast starts, turns, and rapid bursts (Langerhans 2009), so it is possible the origins of their terrestrial crawl kinematics lie in repeated cycles of these behaviors, similarly to *O. maculosus* (Bressman et al. 2018a).

The CCs are greatest (curvature is least) and WAs are least on the boat deck and grass, suggesting lower amplitude movements are sufficient for locomotion on more complex substrates. Smaller amplitude movements may also allow northern snakeheads to have greater SFs on these complex substrates by covering a shorter lateral distance. Overall, northern snakeheads are able to move more effectively and efficiently on natural, complex surfaces like grass, than on artificial, homogenous surfaces like bench liner-covered concrete. Snakeheads are likely able to cover a greater distance and move more quickly in natural, herbaceous riparian zones or grasslands than in manmade, concrete areas with low surface complexity, such as at the edges of artificial canals and on roads. Therefore, natural areas may be at greater risk for terrestrial dispersion of northern snakeheads than urban areas, especially since cover offered by vegetation may reduce their risk of desiccation.

### Conclusions

Overall, northern snakeheads can perform effective overland movements and are in fact the largest species of fish yet described to use a form of axial-appendage-based terrestrial locomotion. Their locomotor performance improves on complex substrates, like grass. Additionally, northern snakeheads emerge from water during physiologically stressful conditions. Snakeheads can also respire in air (Das 1928; Liem 1987; Chew et al. 2003; Courtenay and Williams 2004; Li et al. 2017) and survive out of water for long periods of time while moist (Das 1928). Combined, these traits suggest that northern snakeheads are capable of overland movements between bodies of water, especially when exposed to poor aquatic conditions or during flooding events when fish can be stranded on land or in small ephemeral bodies of water. As an invasive amphibious fish that is currently expanding its range in the United States, it is important to consider their amphibious behaviors and potential to colonize new bodies of water overland when regulating live possession and developing range expansion models and management plans (Orrell and Weigt 2005; Odenkirk and Owens 2007; Love and Newhard 2012, 2018).

## Supplementary Material

obz026_Supplementary_Data
