# Supplementary figures and images for: Emersion and Terrestrial Locomotion of the Northern Snakehead (Channa argus) on Multiple Substrates
Source: Integr Org Biol. 2019 Oct 25;1(1):obz026. doi: 10.1093/iob/obz026 (PMC7671134; doi:10.1093/iob/obz026)

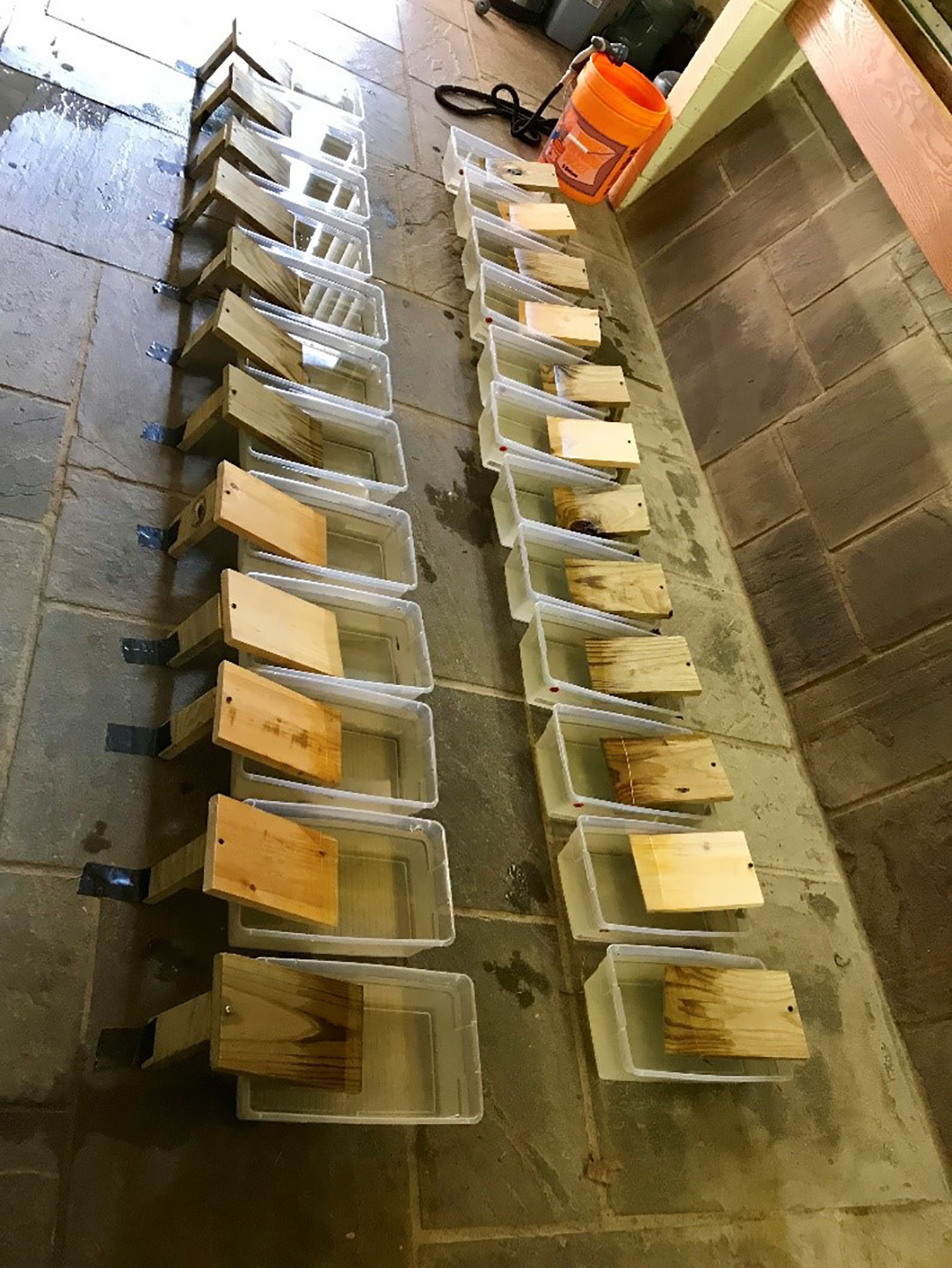

Supplement: obz026_Supplementary_Data [file obz026_supplementary_data.zip › S.Fig1 IOB.jpg]

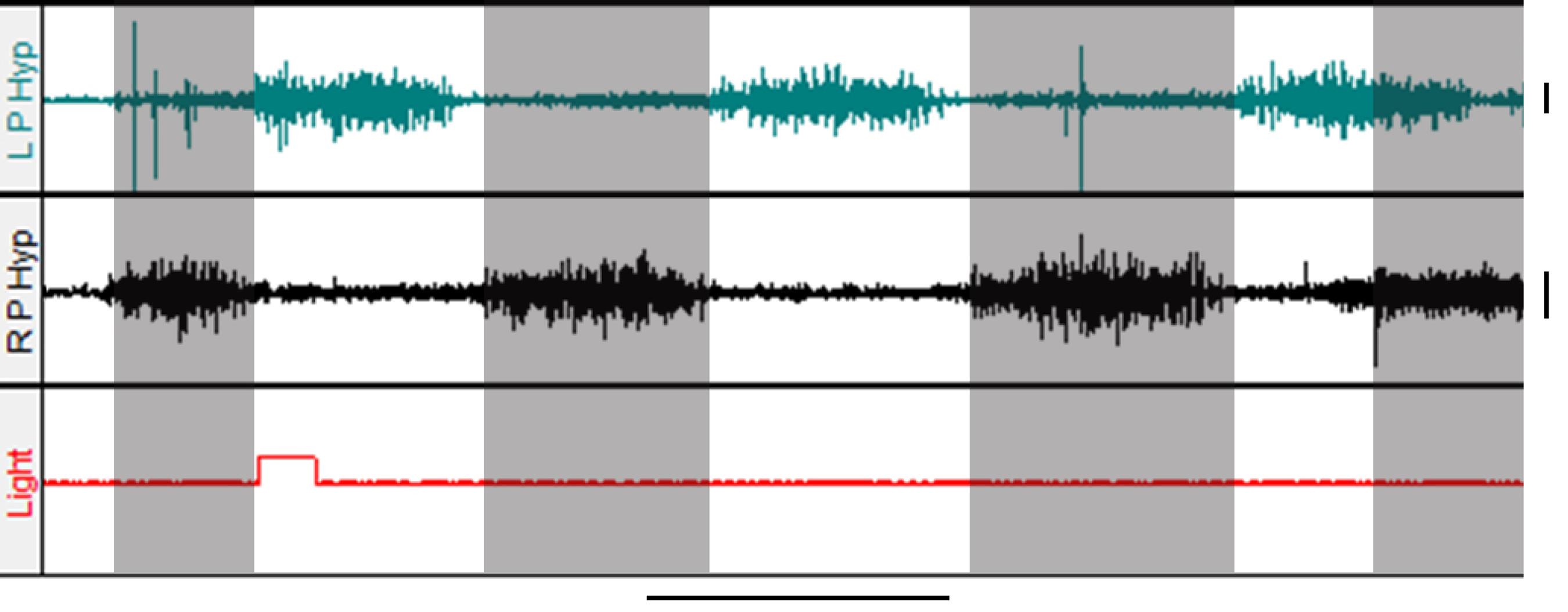

Supplement: obz026_Supplementary_Data [file obz026_supplementary_data.zip › S.Fig3 IOB.jpg]
